# Longitudinal analysis of immune abnormalities in varying severities of Chronic Fatigue Syndrome/Myalgic Encephalomyelitis patients

**DOI:** 10.1186/s12967-015-0653-3

**Published:** 2015-09-14

**Authors:** Sharni Lee Hardcastle, Ekua Weba Brenu, Samantha Johnston, Thao Nguyen, Teilah Huth, Sandra Ramos, Donald Staines, Sonya Marshall-Gradisnik

**Affiliations:** National Centre for Neuroimmunology and Emerging Diseases, 9.22, G40 Griffith Health Institute, School of Medical Science, Griffith University, Parklands Drive, Gold Coast, QLD 4222 Australia

**Keywords:** Chronic Fatigue Syndrome, Longitudinal, Severity, Markers, Natural killer cell

## Abstract

**Background:**

Research has identified immunological abnormalities in Chronic Fatigue Syndrome/Myalgic Encephalomyelitis (CFS/ME), a heterogeneous illness with an unknown cause and absence of diagnostic test. There have been no CFS/ME studies examining innate and adaptive immune cells longitudinally in patients with varying severities. This is the first study to investigate immune cells over 6 months while also examining CFS/ME patients of varying symptom severity.

**Methods:**

Participants were grouped into 18 healthy controls, 12 moderate and 12 severe CFS/ME patients and flow cytometry was used to examine cell parameters at 0 and 6 months.

**Results:**

Over time, iNKT CD62L expression significantly increased in moderate CFS/ME patients and CD56^bright^ NK receptors differed in severe CFS/ME. Naïve CD8^+^T cells, CD8^−^CD4^−^ and CD56^−^CD16^−^ iNKT phenotypes, γδ2T cells and effector memory subsets were significantly increased in severe CFS/ME patients at 6 months. Severe CFS/ME patients were significantly reduced in CD56^bright^CD16^dim^ NKG2D, CD56^dim^CD16^−^ KIR2DL2/DL3, CD94^−^CD11a^−^ γδ1T cells and CD62L^+^CD11a^−^ γδ1T cells at 6 months.

**Conclusions:**

Severe CFS/ME patients differed from controls and moderate CFS/ME patients over time and expressed significant alterations in iNKT cell phenotypes, CD8^+^T cell markers, NK cell receptors and γδT cells at 6 months. This highlights the importance of further assessing these potential immune biomarkers longitudinally in both moderate and severe CFS/ME patients.

**Electronic supplementary material:**

The online version of this article (doi:10.1186/s12967-015-0653-3) contains supplementary material, which is available to authorized users.

## Background

In the immune system, lymphocytes are subject to continual checkpoints, signals and regulation to allow successful cell development, homeostasis and to subsequently prevent illness [1]. Immune responses generated as a result of these signals between the innate and adaptive cells can fluctuate and have a critical influence on the maintenance of physiological homeostasis [1, 2]. Chronic Fatigue Syndrome/Myalgic Encephalomyelitis (CFS/ME) is a heterogeneous illness, varying in severity and nature of onset although research has consistently established immunological abnormalities [3–9].

Reduced Natural Killer (NK) cell cytotoxic activity is the most predominant and consistent outcome of immunological studies in CFS/ME. A number of parameters have also been shown to alter in patients, including T regulatory cells (Tregs), iNKT cells, CD8^+^ T cells and cytokines [6, 8, 10]. Alterations in both innate and adaptive immune cells reflect the extent of immune dysregulation in CFS/ME which may potentially be linked to the illness pathomechanism or contribute to future diagnostic methods.

Longitudinal studies of CFS/ME have also demonstrated consistently reduced NK cell activity while there was variation in cytokine levels over time. It appears that longitudinal examination of immune cells in CFS/ME may allow an assessment of consistent immune parameters as potential biomarkers for the illness [2, 11]. This research further investigates immunological markers of the innate and adaptive immune system at 0 and 6 months in moderate and severe CFS/ME patients.

## Methods

### Participants

This research was granted ethical approval after review by the Griffith University Human Research Ethics Committee (GU Ref No: MSC/23/12/HREC).

Participants previously recruited from Queensland and New South Wales areas of Australia were again approached for this follow-up study. All assessments were taken at 0 and 6 months. Participants were between 20 and 65 years old and CFS/ME patients had the illness for a period of at least 6 months prior to the study. CFS/ME was defined based on the 1994 Fukuda criteria in the absence of a biomarker or diagnostic test for the illness. CFS/ME patients were identified as either moderate or severe and these groups were confirmed using an extensive questionnaire to assess symptomatology, health status, quality of life, severity and mobility in all participants [10]. Participants were excluded if they were previously diagnosed with an autoimmune disorder, psychosis, heart disease or thyroid-related disorders or if they were pregnant, breast feeding, smoking, or experiencing symptoms of CFS/ME that did not conform to the Fukuda criteria for CFS/ME.

Participants (n = 42) in the follow up study included moderately (n = 12) or severely (n = 12) affected CFS/ME patients as well as an age and sex matched non-fatigued control group (n = 18). The severe CFS/ME group were housebound and the Fatigue Severity Scale (FSS), Dr Bell’s Disability Scale, the FibroFatigue Scale and the Karnofsky Performance Scale (KPS) were assessed in all participant groups as a determinant of severity [10, 12].

### Sample preparation

A non-fasting blood sample of 50 mL was collected from the antecubital vein of participants into lithium heparinised and ethylenediaminetetraacetic acid (EDTA) tubes. Blood was collected between 8:00 and 11:30 am and samples were analysed within 12 h of collection. Initial full blood count assessment was undertaken to determine levels of white blood cell and red blood cell markers.

### Intracellular analysis

Density gradient centrifugation using Ficoll-hypaque (Sigma, St Louis, MO) was used to isolate PBMCs from EDTA whole blood. PBMCs were adjusted to 1 × 10^7^ cells/mL and stained with monoclonal antibodies for T regulatory cell (Treg) phenotypes, NK lytic proteins and CD8 lytic proteins as described [4, 6] (Supplementary Table 1). The Treg phenotypes were assessed as PBMCs were permeablised and fixed with buffers containing diethylene glycol and formaldehyde before being stained with FOXP3. After washing with Phosphate Buffered Saline (PBS) (Gibco Biocult, Scotland), cells were analysed on the flow cytometer (Becton–Dickinson Immunocytometry Systems) where the expression of FOXP3^+^ Tregs was determined on CD4^+^CD25^+^CD127^low^ T cells [6]. NK and CD8 T cell lytic proteins were assessed as previously described [6]. Cells were incubated for 30 min in Cytofix then permwash was added. Perforin, granzyme A and granzyme B monoclonal antibodies were added to cells and incubated for 30 min in the dark at room temperature. Cells were then washed and analysed on the flow cytometer where perforin, granzyme A and granzyme B expression was measured in NK and CD8 T cells [6].

### NK phenotype and receptors analysis

Natural killer cells were isolated from whole blood cells using a negative selection system RosetteSep Human Natural Killer Cell Enrichment Cocktail (StemCell Technologies, Vancouver, BC). Isolated NK cells were labelled with CD56, CD16, CD3 (BD Biosciences, San Diego, CA) and monoclonal antibodies for KIR receptors (Additional file 1: Table S1) (Miltenyi Biotec). Cells were analysed on the flow cytometer (Becton–Dickinson Immunocytometry Systems) where NK cells were gated using CD56, CD16 and CD3 antibodies (Additional file 1: Table S1) [4].

### Whole blood analysis

Appropriate antibodies (Additional file 1: Table S1) were added to whole blood samples and incubated for 30 min. Following which cells were lysed, washed, fixed and analysed on the flow cytometer. iNKT, DC, B, γδ T and CD8^+^ T cell phenotypes were assessed using appropriate antibodies (Additional file 1: Table S1) and gating strategies on the flow cytometer (Additional file 1: Table S1) [13].

### Data and statistical analysis

All statistical analysis was performed using SPSS statistical software version 22.0. Paired *t* tests were used to examine changes in each immune parameter between 0 and 6 months for each of the groups. A one-way repeated measures analysis of variance (ANOVA) with time as a within-subject factor and group as a between-subject factor was used to assess the interaction of time and group. Results were classified as statistically significant at an alpha criterion of *p* < 0.05 if there were significant differences between groups over time.

The 6 month single time point analysis was assessed among the three participant groups (control, moderate CFS/ME and severe CFS/ME) based on the distribution. If normally distributed, the analysis of variance test (ANOVA) was used. Shapiro–Wilk and Kruskal–Wallis test of independent variables based on rank sums to determine the magnitudes of group differences was used if data was not normally distributed. The Bonferroni Post Hoc or Mann–Whitney *U* tests determined *p* values of significance for parametric and non-parametric data respectively, with statistical significance set at an alpha criterion at *p* < 0.05. Clinical data are presented as mean ± SD and immunological data are represented using mean ± SEM. Extreme outliers were identified using an SPSS boxplot and handled by eliminating particular data points from the analysis [14].

## Results

### Patient characteristics

The participant ages (mean ± SD) for the control (n = 18), moderate CFS/ME (n = 12) and severe CFS/ME (n = 12) patient groups were 41.94 ± 10.76, 44.73 ± 12.90 and 41.27 ± 10.05 respectively, with no statistically significant differences (*p* < 0.05) in age between the groups (Table 1). Gender distribution was also not significantly different between the groups as they were all predominantly female with control, moderate CFS/ME and severe CFS/ME groups having 72, 67 and 83 % female participants, respectively (Table 1).

**Table 1:** Participant characteristics including age and gender for control, moderate CFS/ME and severe CFS/ME participant groups

	Control (n = 18)	Moderate (n = 12)	Severe (n = 12)	*p* value
Age in years *(mean* *± * *SD)*	41.94 ± 10.76	44.73 ± 12.90	41.27 ± 10.05	0.540
Gender (% Female)	72 %	67 %	83 %	0.566

Age data is represented as mean ± SD and gender is represented as percentage of group which that is female in control (n = 18), moderate CFS/ME (n = 12) and severe CFS/ME (n = 12) groups. There were no significant differences in age or gender within the research groups. *CFS/ME* Chronic Fatigue Syndrome/Myalgic Encephalomyelitis, *SD* standard deviation

**Fig. 1:**
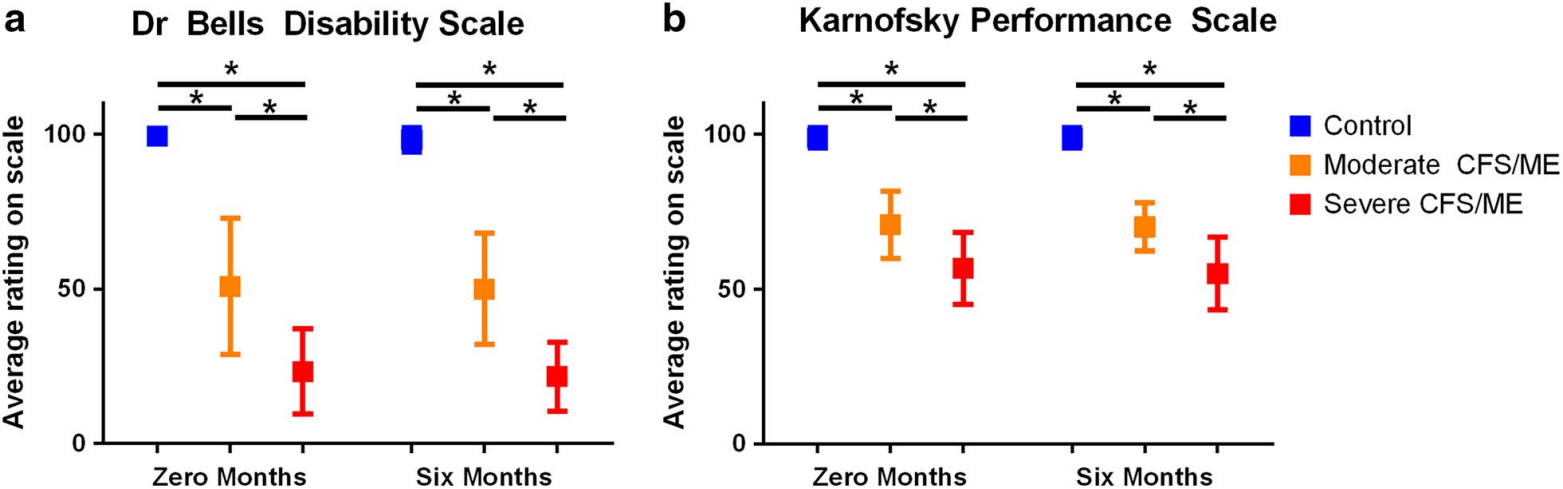
Average severity scale scores at 0 and 6 months for control, moderate and severe CFS/ME patients. **a** Dr Bells Disability Scale scores for each group, represented as a score between 0 and 100. **b** Karnofsky Performance Scale scores for each group, represented as a score between 0 and 100. All data is presented as mean ± SEM where statistical significance was accepted as *p* < 0.05. *CFS/ME* Chronic Fatigue Syndrome/Myalgic Encephalomyelitis, *SEM* standard error of the mean

All CFS/ME patients in the moderate and severe CFS/ME groups satisfied the 1994 Fukuda criteria for CFS/ME as those who did not were excluded from the study. According to the FibroFatigue Scale, all CFS/ME patients scored significantly worse than the control group except in relation to ‘sadness’ which had no differences in scores between any participant groups. There was no statistically significant difference between moderate and severe CFS/ME patients in the FibroFatigue Scales (data not shown). Dr Bells Disability scale (DRS) and the KPS were significantly different between all groups, with severe CFS/ME patients scores being further worsened significantly compared to moderate CFS/ME (Fig. 1).

### No change to intracellular parameters

There were no significant differences between any of the groups and between 0 and 6 months for Tregs, NK or CD8^+^ T cell lytic proteins.

### No change to whole blood phenotypes

This research found no significant differences in DC or B cell phenotypes between any of the groups or between 0 and 6 months.

### iNKT cells

**Fig. 2:**
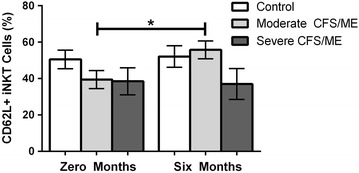
iNKT cell expression of CD62L in control, moderate and severe CFS/ME patients at 0 and 6 months. iNKT cells expressing CD62L as a percentage of total iNKT cells. Data is presented as mean ± SEM where statistical significance was accepted as *p* < 0.05. *CFS/ME* Chronic Fatigue Syndrome/Myalgic Encephalomyelitis, *SEM* standard error of the mean

Between 0 and 6 months, iNKT cells expressing CD62L were significantly increased at 6 months in moderate CFS/ME patients (*p* = 0.004) (Fig. 2).

**Fig. 3:**
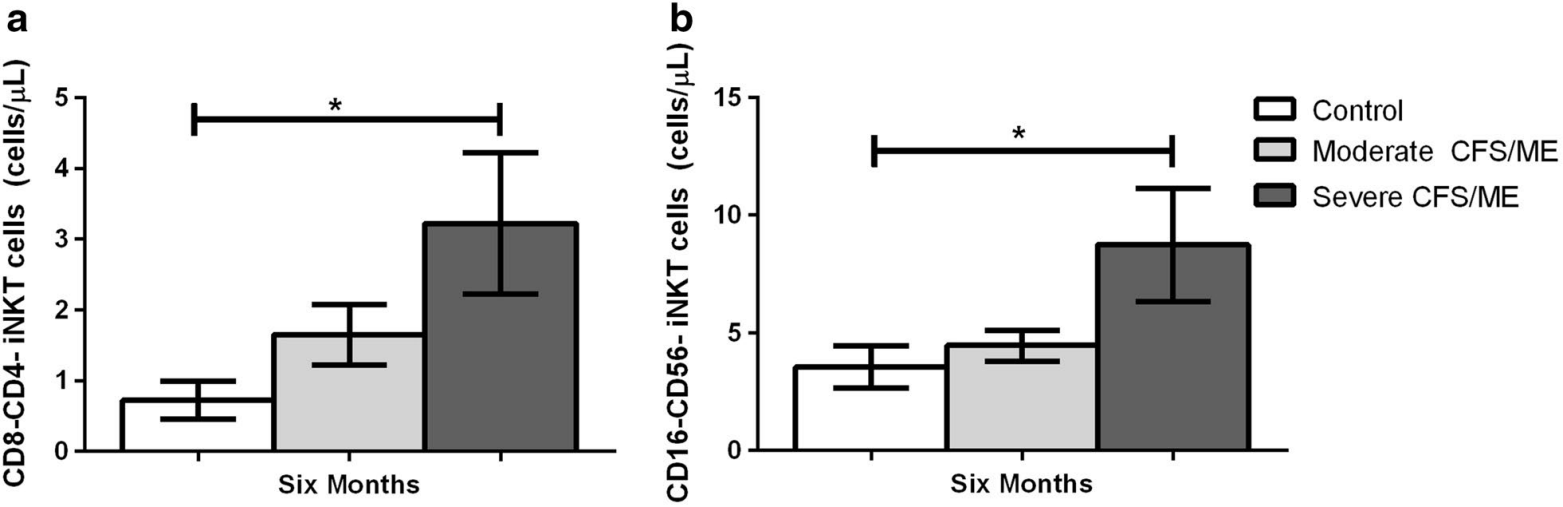
iNKT cell expression profile in control, moderate and severe CFS/ME patients. **a** iNKT cells expressing a CD8^−^CD4^−^ phenotype as a number of total iNKT cells (cells/μL). **b** iNKT cells expressing a CD56^−^CD16^−^ phenotype as a number of total iNKT cells (cells/μL). Data is shown as mean ± SEM where statistical significance was accepted as *p* < 0.05. *CFS/ME* Chronic Fatigue Syndrome/Myalgic Encephalomyelitis, *SEM* standard error of the mean

At the 6th month, CD8^−^CD4^−^ and CD56^−^CD16^−^ iNKT cells were significantly increased in severe CFS/ME compared to controls (*p* = 0.024 and 0.030) (Fig. 3).

### KIRs

**Fig. 4:**
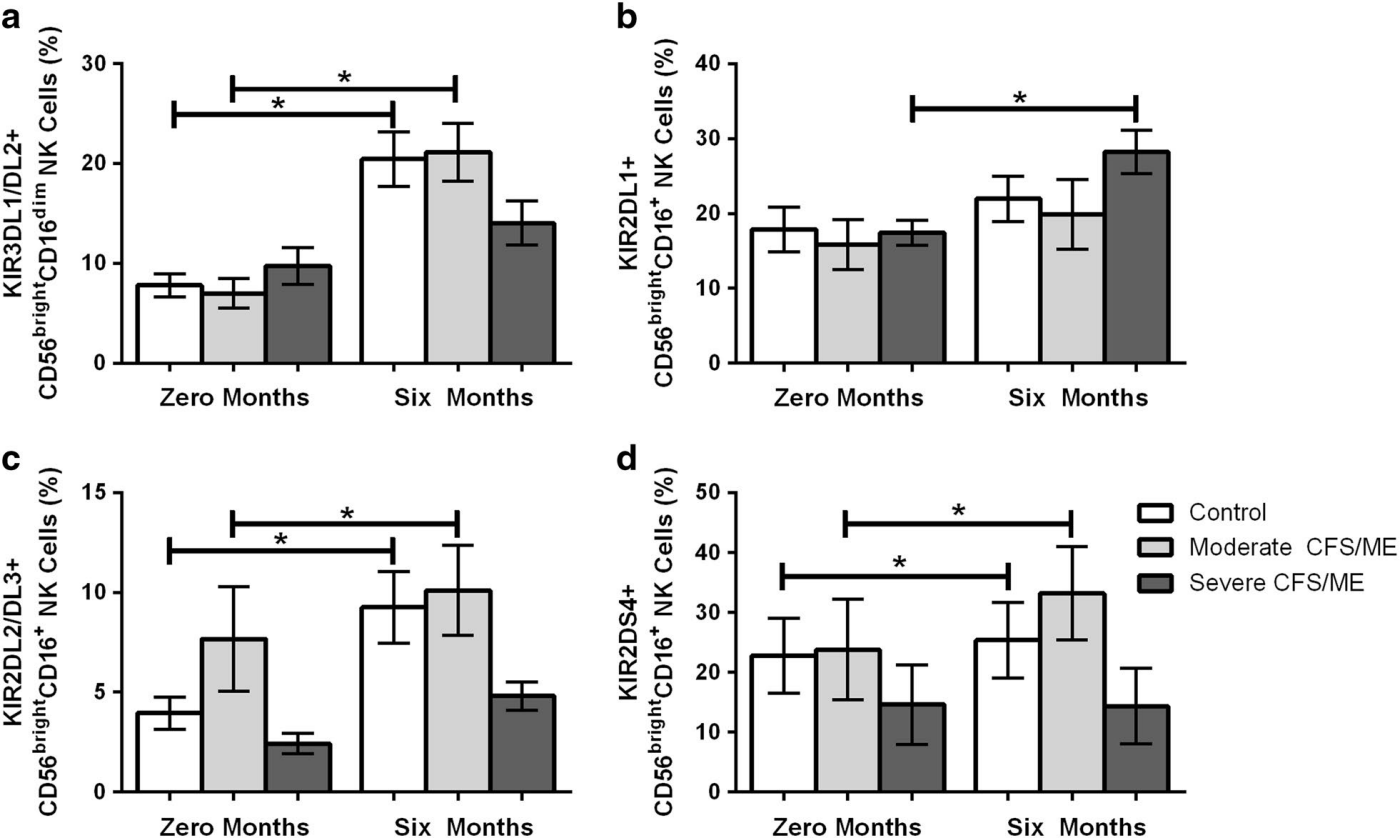
Alterations in CD56^bright^ NK cell receptors between 0 and 6 months in control, moderate and severe CFS/ME patients. **a** Percentage of CD56^bright^CD16^dim^ NK cells expressing KIR3DL1/DL2. **b** Percentage of total CD56^bright^CD16^+^ NK cells expressing KIR2DL1. **c** Percentage of CD56^bright^CD16^+^ NK cells expressing KIR2DL2/DL3. **d** Percentage of CD56^bright^CD16^+^ NK cells expressing KIR2DS4. Data is shown as mean ± SEM where statistical significance was accepted as *p* < 0.05. *NK* Natural Killer, *CFS/ME* Chronic Fatigue Syndrome/Myalgic Encephalomyelitis, *SEM* standard error of the mean

CD56^bright^CD16^dim^ NK cells expressing KIR3DL1/DL2 were significantly increased in controls and moderate CFS/ME patients after 6 months (*p* < 0.000 and 0.004) (Fig. 4a). CD56^bright^CD16^+^ NK cells expressing KIR2DL1 were significantly increased in severe CFS/ME patients after 6 months (*p* = 0.011) (Fig. 4b). CD56^bright^CD16^+^ NK cells expressing KIR2DL2/DL3 were significantly increased in controls and moderate CFS/ME patients after 6 months (*p* = 0.018 and 0.049) (Fig. 4c). CD56^bright^CD16^+^ NK cells expressing KIR2DS4 were also significantly increased in controls and moderate CFS/ME patients after 6 months (*p* = 0.038 and 0.023) (Fig. 4d).

**Fig. 5:**
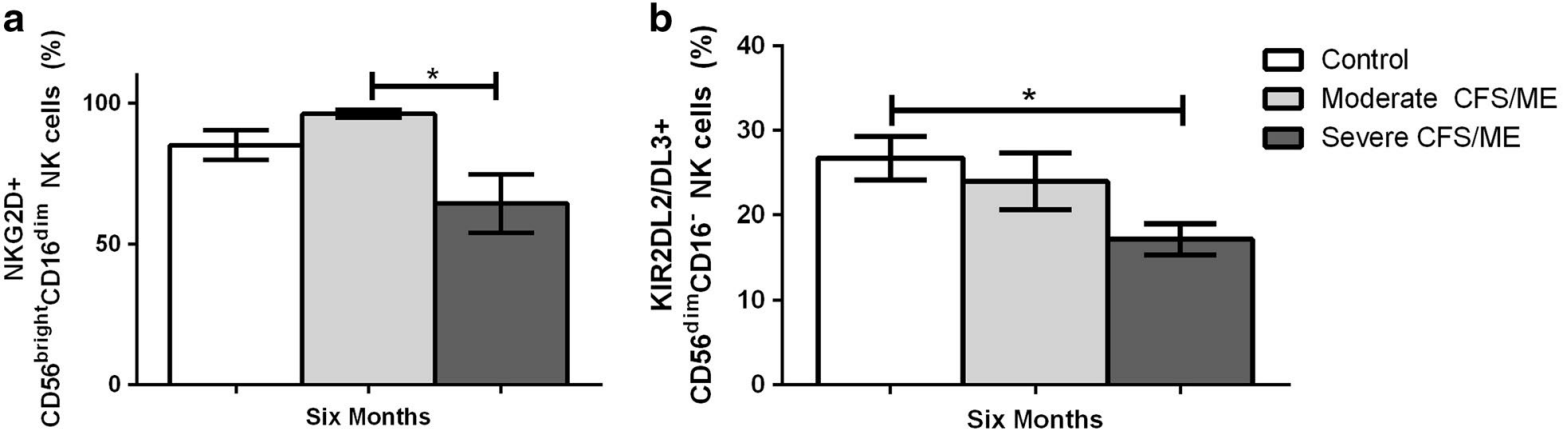
NK cell receptors in control, moderate and severe CFS/ME participant groups. **a** Percentage of total CD56^bright^CD16^dim^ NK cells expressing the receptor NKG2D. **b** Percentage of total CD56^dim^CD16^−^ NK cells expressing the receptor KIR2DL2/DL3. Data is shown as mean ± SEM where statistical significance was accepted as *p* < 0.05. *NK* Natural Killer, *CFS/ME* Chronic Fatigue Syndrome/Myalgic Encephalomyelitis, *SEM* standard error of the mean

At 6 months, CD56^bright^CD16^dim^ NK cells expressing NKG2D were significantly reduced in severe CFS/ME compared to moderate CFS/ME patients (*p* = 0.014) (Fig. 5a). Also at 6 months, KIR2DL2/DL3 expression in CD56^dim^CD16^−^ NK cells was significantly reduced in severe CFS/ME patients compared to controls (*p* = 0.045) (Fig. 5b).

### CD8 T cells

**Fig. 6:**
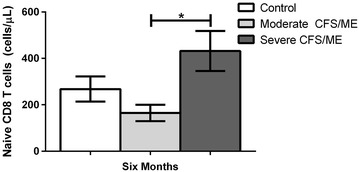
Increased naïve CD8^+^ T cells in the severe CFS/ME participant group. Number of total CD8^+^ T cells (cells/μL) of the naïve CD8^+^ T cell subset in controls, moderate CFS/ME and severe CFS/ME. Data is shown as mean ± SEM where statistical significance was accepted as *p* < 0.05. *CFS/ME* Chronic Fatigue Syndrome/Myalgic Encephalomyelitis, *SEM* standard error of the mean

At 6 months, naïve CD8 T cells were significantly increased in severe CFS/ME patients compared to moderate CFS/ME patients (*p* = 0.041) (Fig. 6).

### γδ T cells

**Fig. 7:**
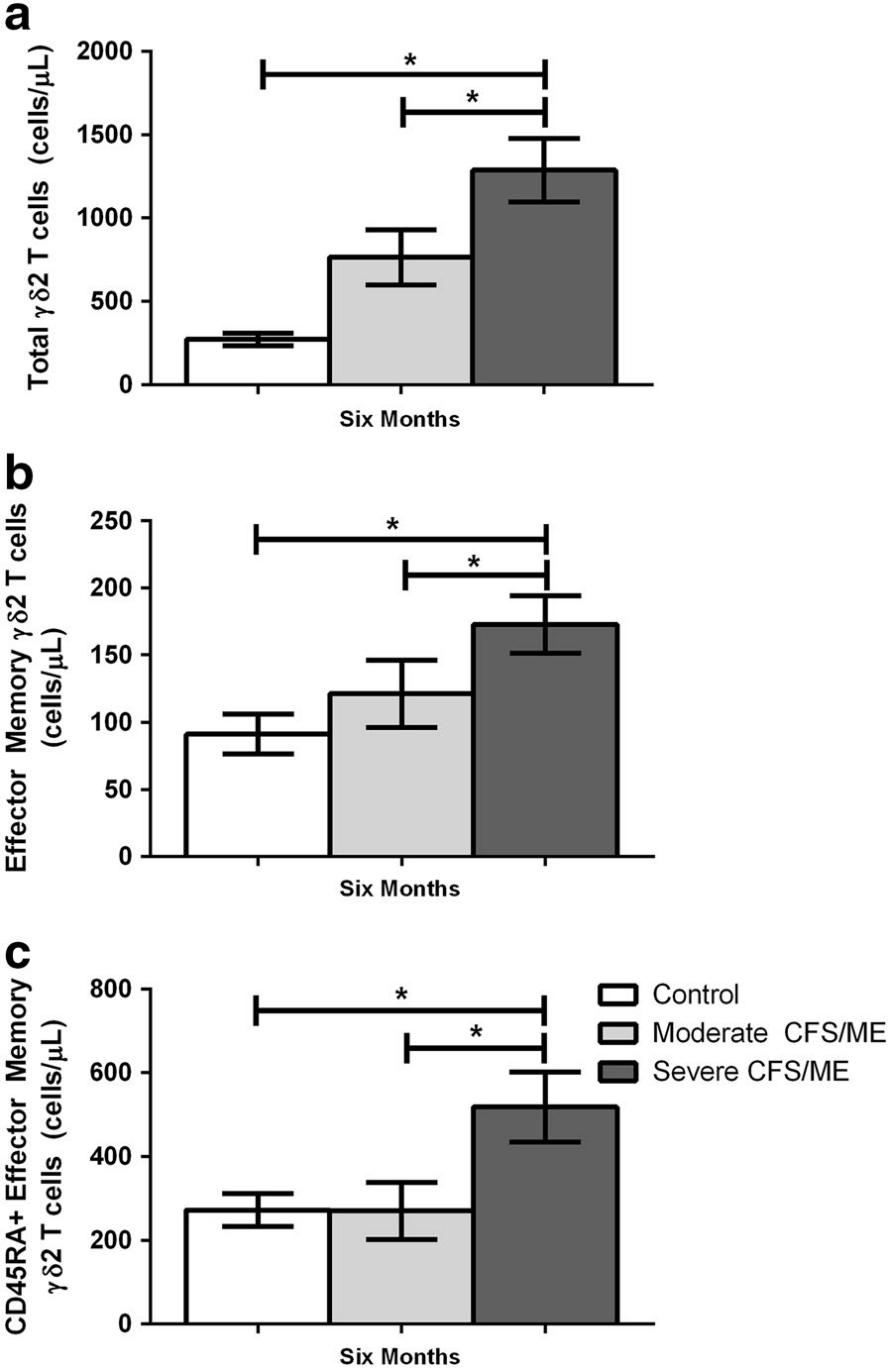
Alterations in γδ2 T cell phenotypes in control, moderate CFS/ME and severe CFS/ME patients. **a** Total number of γδ2 T cells expressed as total cells/μL. **b** Total number of γδ2 T cells (cells/μL) expressing the effector memory cell phenotype. **c** Total number of γδ2 T cells (cells/μL) expressing the CD45RA^+^ effector memory cell phenotype. Data is shown as mean ± SEM where statistical significance was accepted as *p* < 0.05. *CFS/ME* Chronic Fatigue Syndrome/Myalgic Encephalomyelitis, *SEM* standard error of the mean

At the 6 months, total γδ2 T cells were significantly increased in severe CFS/ME compared to controls and moderate CFS/ME patients (*p* = 0.035 and 0.034) (Fig. 7a). At 6 months, γδ2 effector memory and CD45RA^+^ effector memory T cells were also significantly increased in the severe CFS/ME patient group compared to controls and moderate CFS/ME patients respectively (*p* = 0.003, 0.013 and 0.017, 0.032) (Fig. 7b, c).

**Fig. 8:**
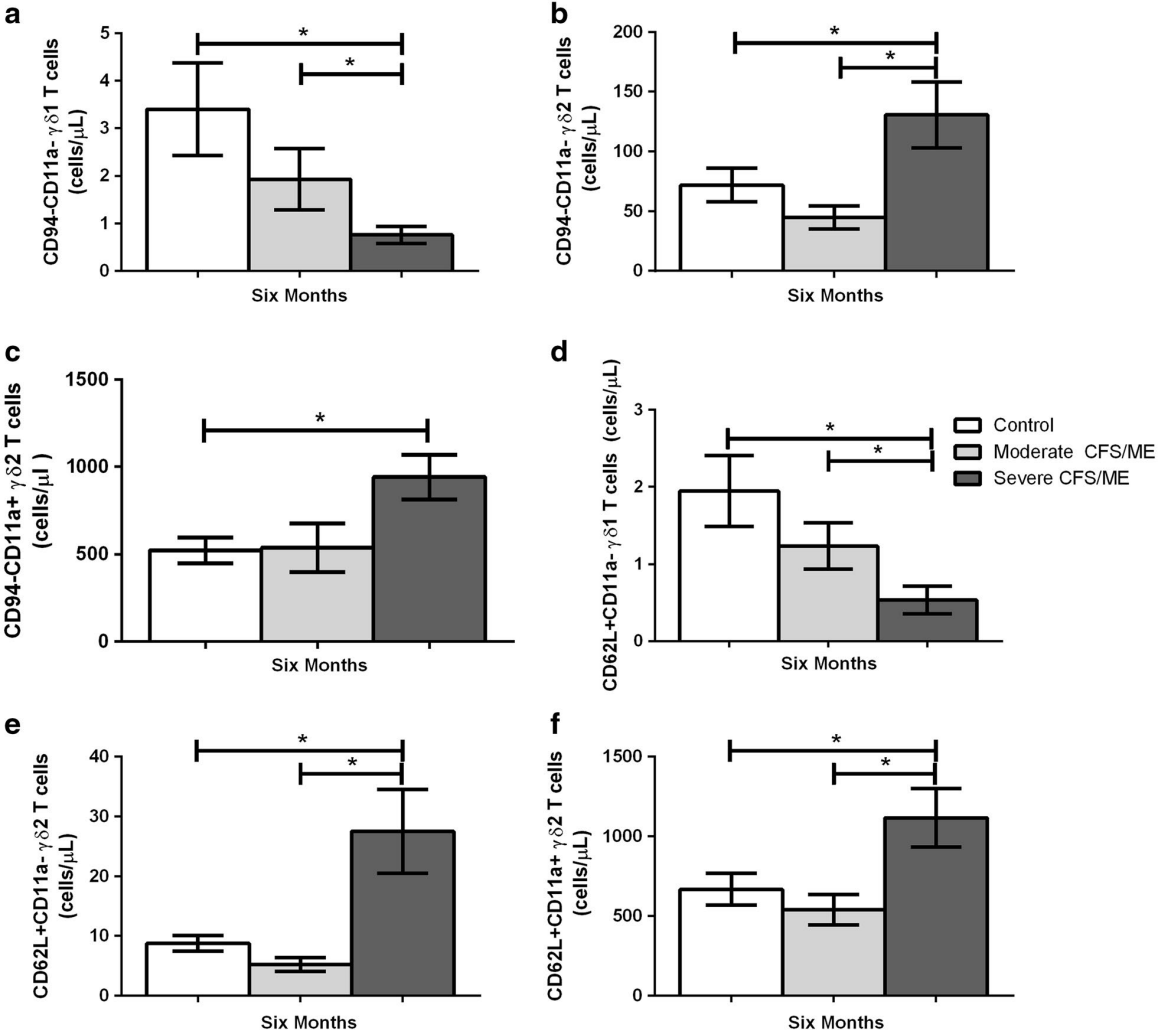
Profile of γδ1 and γδ2 T cell expression of receptors and adhesion molecules in control, moderate CFS/ME and severe CFS/ME patients. **a** γδ1 T cells with the expression CD94^−^CD11a^−^, as a number of total γδ1 T cells (cells/μL). **b** γδ2 T cells with the expression CD94^−^CD11a^−^, as a total number of γδ2 T cells (cells/μL). **c** γδ2 T cells with the expression CD94^−^CD11a^+^, as a total number of γδ2 T cells (cells/μL). **d** γδ1 T cells expressing CD62L^+^CD11a^−^ as a total number of γδ1 T cells (cells/μL). **e** γδ2 T cells expressing CD62L^+^CD11a^−^, as a total number of γδ2 T cells (cells/μL). **f** γδ2 T cells with the expression CD62L^+^CD11a^+^, as a total number of γδ2 T cells (cells/μL). Data is shown as mean ± SEM where statistical significance was accepted as *p* < 0.05. *CFS/ME* Chronic Fatigue Syndrome/Myalgic Encephalomyelitis, *SEM* standard error of the mean

At 6 months, γδ1 T cells in the severe CFS/ME group displayed significantly lower CD94^−^CD11a^−^ expression when compared to the control and moderate CFS/ME group (*p* = 0.018 and 0.047) (Fig. 8a). At 6 months, CD94^−^CD11a^−^ expression in γδ2 T cells of severe CFS/ME patients was significantly higher than controls and moderate CFS/ME (*p* = 0.019 and 0.005) (Fig. 8b). The severe CFS/ME group also had significantly higher CD94^−^CD11a^+^ expression on γδ2 T cells compared to controls (*p* = 0.025) in the 6 month (Fig. 8c).

At the 6th month, γδ1 T cells expression of CD62L^+^CD11a^−^ was significantly reduced in severe CFS/ME compared to both controls and moderate CFS/ME (*p* = 0.013 and 0.023) (Fig. 8d). At 6 months, γδ2 T cells expression of CD62L^+^CD11a^−^ as well as CD62L^+^CD11a^+^ was significantly increased in the severe CFS/ME group compared to controls and moderate CFS/ME patients (*p* = 0.002, 0.001 and 0.045, 0.018 respectively) (Fig. 8e, f).

## Discussion

The present study examined innate and adaptive immune cells at 0 and 6 months to investigate longitudinal changes in moderate and severe CFS/ME. Severe CFS/ME patients displayed significant NK cell receptor differences over time when compared to controls and moderate CFS/ME. At the 6th month, severe CFS/ME patients also demonstrated significant alterations in iNKT cell phenotypes, CD8^+^ T cell markers, NK cell receptors and γδ T cells compared with the control and/or moderate CFS/ME patients.

Our study demonstrated immunological variation over time as there were differences between participant groups between 0 and 6 months. iNKT cells had not previously been examined in CFS/ME and the current study found expression of CD62L was significantly increased in moderate CFS/ME patients between 0 and 6 months. The function of CD62L in iNKT cells is not known although this may suggest variation in iNKT cell markers or adhesion over time in CFS/ME. The CD56^bright^ NK cell subset also varied between participant groups over time, particularly in the severe CFS/ME patients. CD56^bright^CD16^dim^ NK cells expressing KIR3DL1/DL2 and CD56^bright^CD16^+^ NK cells expressing KIR2DS4 and KIR2DL2/DL3 were significantly increased after 6 months in controls and moderate CFS/ME patients, while severe CFS/ME patients showed significantly increased CD56^bright^CD16^+^ NK cells expressing the KIR2DL1 receptor after 6 months. This research showed changes in NK receptors over time notably in CD56^bright^ NK cells. CD56^bright^ NK cells form around 10 % of total peripheral NK cells and are the primary producers of NK cell-derived cytokines, particularly IFN-γ, TNF-β, macrophage colony-stimulating factor (M-CSF), IL-10 and IL-13 during an innate immune response [15, 16]. Previously, peripheral levels of IL-10 and IFN-γ were shown to be significantly increased and longitudinal analysis has shown the CD56^bright^CD16^−^ NK cell phenotype to be decreased over time in CFS/ME patients [2]. The current study potentially suggests that the alterations in CD56^bright^ NK cell subsets may be influencing cytokine production over time in CFS/ME. Cytokine imbalances between proinflammatory cytokines or cytokine inhibitors may play a role in the initiation of a number of diseases, particularly Th1/Th2 cytokine shifts which have been used to explain immunological disease pathogenesis [17].

Natural killer cell receptors are particularly important in CFS/ME as reduced NK cell cytotoxic activity is one of the most consistent markers of the illness [2–4, 6–8, 10, 18]. NK cell cytotoxic activity can be regulated to by NKG2D, which is an activating receptor that has previously been significantly elevated in International Consensus Criteria for CFS/ME-defined CFS/ME patients when compared to CFS/ME patients defined using the 1994 Fukuda definition [5]. CD94 is a NK receptor which is dependent on NKG2 protein association and has also been significantly increased in CD56^dim^CD16^−^ NK cells in CFS/ME patients in previous research [10]. Our study found significantly reduced NKG2D expression in CD56^bright^CD16^dim^ NK cells in severe CFS/ME compared to moderate CFS/ME at the 6th month. Therefore, reduced NKG2D may be associated with the reduced NK cell cytotoxic activity previously shown in severe CFS/ME patients compared to moderate CFS/ME patients [4, 10]. Previous research has also suggested that impairment of NK cell cytolytic function may be derived in part by reduced activating NK cell receptors, such as NKG2D [19].

KIR2DL2/DL3 is an inhibitory receptor that has been previously reduced in severe CFS/ME compared to moderate CFS/ME patients [10]. The current study supports previous findings where KIR2DL2/DL3 expression in CD56^dim^CD16^−^ NK cells in severe CFS/ME patients was again significantly reduced when compared to controls. This reduction in the inhibitory receptor of CFS/ME patients may be a result of a larger regulatory response to the reduced NK cell cytotoxic activity that is shown in the illness, particularly as CD56^dim^ NK cells are highly cytotoxic [4, 15].

Significantly raised naïve CD8^+^ T cell numbers at the 6th month of this research in severe CFS/ME patients may be promoting the ability of these severely affected patients to develop an immune response against novel antigens and lower the susceptibility of infections [20]. CD8^+^ T cells are also responsible for cytotoxic activities and have previously shown significantly reduced activity in CFS/ME patients [6]. In contrast, CFS/ME patients have also previously been associated with CD8^+^ T cell immune activation, a reduced level of CD8^+^ suppressor T cells and an increase in CD8^+^ cytotoxic T cells [21]. Therefore the current study validates previous research where significantly enhanced CD8^+^ T cell activation and CD8^+^ T cell numbers were found in CFS/ME patients [8, 21].

There is little research on iNKT cells in CFS/ME patients, although one study has shown significantly elevated iNKT cell numbers in severe CFS/ME patients, reduced CD8CD4, CD8aCD4 phenotypes in moderate CFS/ME, increased CD56CD16 and CCR7SLAM phenotypes in severe CFS/ME compared to both moderate CFS/ME patients and controls [10]. The present study again found significantly increased iNKT cells expressing CD56^−^CD16^−^ in severe CFS/ME patients at the 6th month. The function of CD56 and CD16 on iNKT cells is unknown [13] however, altered expression of these markers on NK cell phenotypes is often shown in CFS/ME patients [3, 5, 6, 10, 21, 22]. The CD8^−^ CD4^−^ subset of iNKT cells is primarily responsible for cytotoxic activities and was previously reduced in moderate CFS/ME patients [10]. The present study has shown a significant increase in CD8^−^ CD4^−^ iNKT cells in severe CFS/ME patients, suggesting a possible regulatory mechanism where cytotoxic activities may be enhanced in iNKT cells as a regulatory response to the reduced cytotoxic activity that has been consistently documented in NK cells and CD8^+^ T cells of CFS/ME patients [3, 4, 6, 8, 10].

The present study found significantly increased overall numbers of γδ2 T cells in severe CFS/ME at the 6th month. As γδ T cells are sentinel cells with cytotoxic properties, this may suggest an activation as an immune response to bacterial infection, wound repair, antigen presentation or immunoregulation [23]. Significantly enhanced numbers of effector memory and CD45RA^+^ effector memory γδ2 T cells also in severe CFS/ME patients suggests that they have greater potential for cytotoxic activity, tissue homing and target recognition [10, 23]. Effector memory phenotypes of γδ T cells exhibit NK-like functions, detecting major histocompatibility complex (MHC) expression and undergoing cytotoxic activities following cytokine directed proliferation and regulatory pathways [23–25]. Interesting, both effector memory and CD45RA^+^ effector memory T cell phenotypes are preferentially mobilized during adrenergic stimulation, suggesting severe CFS/ME patients’ immune responses may be enhanced similar to a situation of psychological stress [23]. There may potentially be a homeostatic mechanism taking place in severe CFS/ME patients, leading to greater immune activation, similarly to that also shown in CD8^+^ T cells and Tregs in CFS/ME [2, 6, 7, 26].

CD94^−^CD11a^−^ expression was significantly reduced in severe CFS/ME patients in γδ1 T cells and significantly increased in severe CFS/ME patients in γδ2 T cells. CD94 is a surface molecule with NK-like abilities, important in MHC expression detection and high cytotoxic activities while CD11a is an adhesion molecule which aids migration to inflammatory sites [23]. γδ2 T cells expressing CD94^−^CD11a^+^ were also significantly increased in severe CFS/ME patients, suggesting that the majority of γδ T cells in these patients may have improved adhesion and migration to sites of inflammation [23]. γδ1 and γδ2 T cells also showed variation in CD62LCD11a expression, as severe CFS/ME patients demonstrated significantly reduced CD62L^+^CD11a^−^ γδ1 T cells as well as significantly increased CD62L^+^CD11a^−^ γδ2 T cells. CD62L^+^CD11a^+^ expression was increased in γδ2 T cells of severe CFS/ME patients, again potentially suggesting severe patients may have an enhanced immune activation and an increased adhesive and migratory ability compared to moderate CFS/ME and controls [23]. The alternative expression of these markers in γδ1 and γδ2 T cells may be a result of the differing γδ T cells phenotypes while γδ1 T cells are mainly present in epithelial tissues and low levels in the bloodstream and γδ2 T cells represent most of circulating γδ T cells [27].

## Conclusions

This research was the first to assess innate and adaptive immune cells over time in moderate and severe CFS/ME patients. Severe CFS/ME patients had significantly altered NK cell receptors over time in comparison to moderate CFS/ME patients and controls. Severe CFS/ME patients also expressed significant changes in iNKT cell phenotypes, CD8^+^ T cell markers, NK cell receptors and γδ T cells compared to the control and/or moderate CFS/ME patients at 6 months. This research highlighted the importance of longitudinally assessing varying severities of CFS/ME patients to further examine variation in illness severity and consistency of potential immune abnormalities that have been shown. This research may also contribute to further understanding CFS/ME and potentially assist in leading to a diagnostic test based on distinct immunological markers.

## Additional file

**Additional file 1.** Monoclonal antibody combinations used for gating on the flow cytometer to identify various innate and adaptive immune cells and phenotypes.
