# Supplementary material for: Longitudinal analysis of immune abnormalities in varying severities of Chronic Fatigue Syndrome/Myalgic Encephalomyelitis patients
Source: J Transl Med. 2015 Sep 14;13:299. doi: 10.1186/s12967-015-0653-3 (PMC4568602; doi:10.1186/s12967-015-0653-3)
Supplement: Supplementary file 1 — Additional file 1. Monoclonal antibody combinations used for gating on the flow cytometer to identify various innate and adaptive immune cells and phenotypes. [file 12967_2015_653_MOESM1_ESM.docx]

**Additional Table 1:** **Monoclonal antibody combinations used to identify various innate and adaptive immune cells and phenotypes.**

| Cell | Measured Phenotype | Monoclonal Antibody Marker Combinations |
| --- | --- | --- |
| NK Cells | **Phenotypes**:  **KIRs**:  **Lytic Proteins:** | CD3^-^CD56^+/-^CD16^+/-^  KIR2DL1 (CD158a), KIR3DL1 (CD158e), KIR2DL2/DL3 (CD158b), KIR2DS4 (CD158i), KIR2DL1/DS1 (CD158a/h), KIR3DL1/DL2 (CD158e/k), KIR2DL5 (CD158f), NKG2D (CD314), NKG2 (CD94)  CD3^-^CD56^+/-^CD16^+/-^Perforin^+^, CD3^-^CD56^+/-^CD16^+/-^GranzymeA^+^, CD3^-^CD56^+/-^CD16^+/-^GranzymeB^+^ |
| iNKT Cells | **Phenotypes:** | 6B11^+^CD3^+^CD8^+/-^CD4^+/-^, 6B11^+^CD3^+^CD8a^+/-^CD4^+/-^6B11^+^CD3^+^CD45RO^+/-^CD28^+/-^, 6B11^+^CD3^+^CD45RA^+/-^CD27^+/-^, 6B11^+^CD3^+^CCR7^+/-^SLAM^+/-^, 6B11^+^CD3^+^CD56^-/+^CD16^-/+^, 6B11^+^CD3^+^CD62L^-/+^CD11a^-/+^, 6B11^+^CD3^+^CD94^-/+^CD11a^-/+^ |
| CD8 T cells | **Phenotypes:**  **Lytic Proteins:** | CD8^+^CD3^+^CD45RO^+/-^CD27^+/-^, CD8^+^CD3^+^CD45RA^+/-^CD27^+/-^, CD8^+^CD3^+^CCR7^+/-^CCR5^+/-^, CD8^+^CD3^+^CCR5^+/-^CD28^+/-^  CD8^+^CD3^+^Perforin^+^, CD8^+^CD3^+^GranzymeA^+^, CD8^+^CD3^+^GranzymeB^+^ |
| Tregs | **Treg FOXP3^+^:** | CD127^low^CD25^+^CD4^+^FOXP3^+^ |
| γδ T cells | **γδ 1 T cells:**  **γδ 2 T cells:**  **Phenotypes:** | γδ 1^+^CD3^+^CD45RA^+/-^CD27^+/-^  γδ 2^+^CD3^+^CD45RA^+/-^CD27^+/-^  Naïve: γδ^+^CD3^+^CD45RA^+^CD27^+^  Central Memory: γδ^+^CD3^+^CD45RA^-^CD27^+^  Effector Memory: γδ^+^CD3^+^CD45RA^-^CD27^-^  CD45RA^+^ Effector Memory: γδ^+^CD3^+^CD45RA^+^CD27^-^ |
| DCs | **Phenotypes:** | CD14^-^CD16^+^ DCs: Lin2^-^HLA-DR^+^CD16^+^  pDCs: Lin2^-^HLA-DR^+^CD123^+^  mDCs: Lin2^-^HLA-DR^+^CD33^+^ |
| B Cells | **Phenotypes:** | Total: CD19^+^  Memory: CD19^+^ CD27^+^CD38^-^  Plasma: CD19^+^CD138^+^CD27^+^CD38^+^  Plasmablast: CD19^+^CD138^-^CD27^+^CD38^+^  Immature: CD19^+^ CD27^-^CD38^+^ |

Supplementary Table 1 shows the monoclonal antibody combinations used to identify each of the cells and parameters for gating on the flow cytometer.
